# The São Paulo Research Foundation, FAPESP

**DOI:** 10.1186/1753-6561-7-S2-A2

**Published:** 2013-04-04

**Authors:** Carlos Henrique de Brito Cruz

**Affiliations:** 1 São Paulo Research Foundation, São Paulo, SP, Brazil

## 

FAPESP (http://www.fapesp.br/en) is a public foundation, funded by the taxpayer in the State of São Paulo, with the mission to support research projects in higher education and research institutions, in all fields of knowledge. São Paulo has a population of forty million and generates 35% of Brazil’s GNP. The constitution of the State establishes that 1% of all state taxes belong to the foundation and the government transfers these funds monthly. The stability of the funding and the autonomy of the foundation allow for an efficient management of the resources that has had a sizable impact: while São Paulo has 22% of the Brazilian population and 30% of the scientists with a doctorate in the country, the state responds for 52% of the country’s scientific articles published in international journals.

The foundation works in close contact with the scientific community: all proposals are peer reviewed with the help of area panels composed of active researchers. Besides funding research in all fields, the foundation supports large research programs in Biodiversity, Bioenergy, Global Climate Change, and in Neurosciences.

FAPESP invested R$ 939 million (approximately US$ 660 million) in research projects in 2011. One third of this value goes into fellowships for graduate and undergraduate students. About 55% goes into exploratory academic research, mostly fundamental in nature. The remaining 10% is invested into application oriented research, in many cases performed in Small Businesses or in joint research performed by academia and industry. The percentage invested in applied research has been growing in recent years, consistently with the foundation’s mandate to foster the scientific and technological development in the State of São Paulo.

FAPESP maintains cooperation agreements with national and international research funding agencies, higher educational and research institutions and business enterprises. The international cooperation covers a broad range of countries and agencies (http://www.fapesp.br/en/6812) including the UK Research Councils, the Agence Nationale de Recherche (ANR) in France, the Deutsche Forschungsgemeinschaft (DFG) in Germany, and NSF in the U.S.

FAPESP offers many programs to support foreign scientists willing to work in research institutions in the state of São Paulo, Brazil. These include: post-doctoral fellowships (http://www.fapesp.br/en/5427), young investigator awards (http://www.fapesp.br/en/4479) and visiting researcher grants (http://www.fapesp.br/147).

